# Emerging Regulation and Function of Betatrophin

**DOI:** 10.3390/ijms151223640

**Published:** 2014-12-18

**Authors:** Yi-Hsin Tseng, Yung-Hsin Yeh, Wei-Jan Chen, Kwang-Huei Lin

**Affiliations:** 1 Graduate Institute of Biomedical Sciences, College of Medicine, Chang Gung University, Taoyuan 333, Taiwan; E-Mail: akiraest@yahoo.com.tw; 2 First Cardiovascular Division, Chang Gung Memorial Hospital, Taoyuan 333, Taiwan; E-Mails: yys0tw@yahoo.ca (Y.-H.Y.); wjchen@adm.cgmh.com.tw (W.-J.C.)

**Keywords:** betatrophin, C19orf80, chromosome 19 open reading frame 80, RIFL, lipasin, ANGPTL8, TD26, thyroid hormone, irisin, insulin and lipid

## Abstract

Betatrophin, also known as TD26/RIFL/lipasin/ANGPTL8/C19orf80, is a novel protein predominantly expressed in human liver. To date, several betatrophin orthologs have been identified in mammals. Increasing evidence has revealed an association between betatrophin expression and serum lipid profiles, particularly in patients with obesity or diabetes. Stimulators of betatrophin, such as insulin, thyroid hormone, irisin and caloric intake, are usually relevant to energy expenditure or thermogenesis. In murine models, serum triglyceride levels as well as pancreatic cell proliferation are potently enhanced by betatrophin. Intriguingly, conflicting phenomena have also been reported that betatrophin suppresses hepatic triglyceride levels, suggesting that betatrophin function is mediated by complex regulatory processes. However, its precise physiological role remains unclear at present. In this review, we have summarized the current findings on betatrophin and their implications.

## 1. Introduction

Betatrophin, also designated as TD26, re-feeding induced fat and liver (RIFL), lipasin, angiopoietin-like protein 8 (ANGPTL8) and chromosome 19 open reading frame 80 (C19orf80), has been identified and characterized by several groups, and shows promise as a therapeutic agent for metabolic syndrome and Type II diabetes. However, the physiological functions and molecular targets of this protein remain largely unknown. Recent studies have revealed a number of various roles of betatrophin within human and murine models. Here, we provide a review of the gene structure, cellular functions and regulatory mechanisms of betatrophin.

## 2. The Betatrophin Gene

### 2.1. Identification of Betatrophin

The betatrophin protein was initially detected in 2004 as a tumor-associated antigen in patient serum [1]. However, very few subsequent studies focused on further characterization of this novel protein following its identification. In 2012, betatrophin was shown to correlate with the serum triglyceride (TG) level and regulate lipase activity in mouse for the first time [2,3,4]. More recently, Yi *et al*. [5], demonstrated that murine pancreatic cell proliferation is potently activated by β-cell agonists through stimulation of hepatic betatrophin expression. Accumulating data have highlighted the lipid metabolism function of betatrophin.

**Figure 1:**
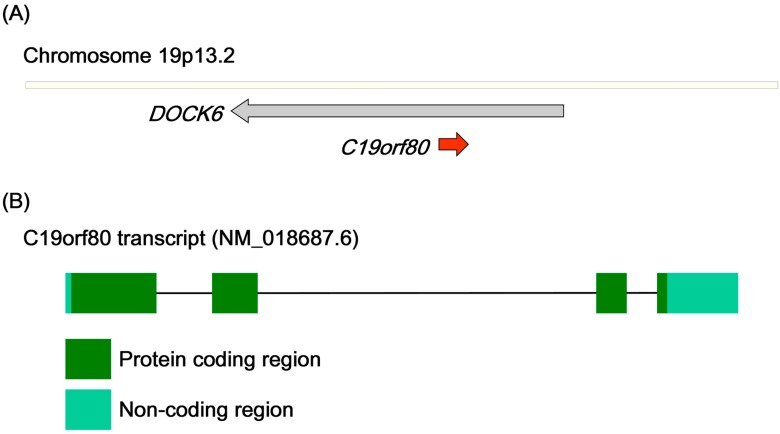
Gene structure of Homo sapiens *C19orf80* (betatrophin). (**A**) The *betatrophin* gene is located on chromosome 19p13.2; (**B**) The human *betatrophin* transcript (NM_018687.6).

**Figure 2:**
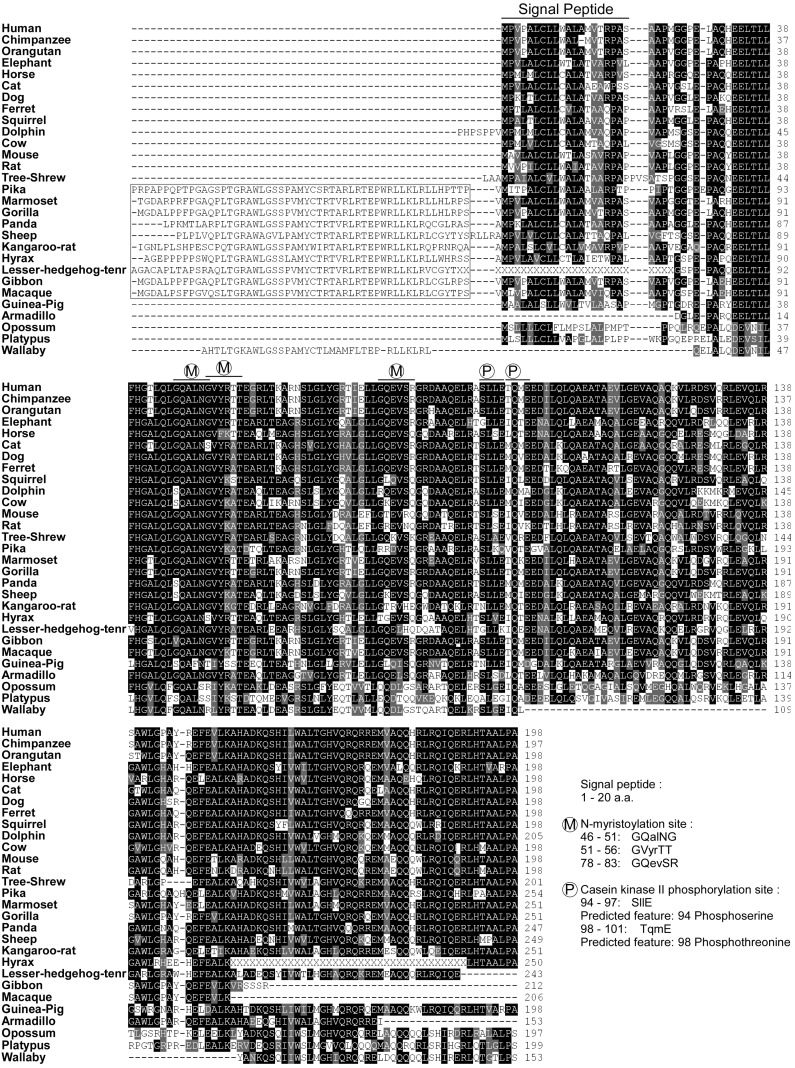
Sequence alignment of betatrophin orthologs in mammals. Amino acid sequences alignment of identified betatrophin orthologs (sequences in black color, identical; grey color, similar). Two suborders, megabat and microbat, are not included for their divergent sequence and thermogenesis regulation. The putative signal peptide and protein modification sites (M; *N*-myristoylation, P; casein kinase phosphorylation, estimated using Pro-site) are indicated. The extra *N*-terminal sequences which conserved in several species are also highlight (from Pika to Macaque, 10 species).

### 2.2. Gene Structure and Predicted Motifs of Betatrophin

The *betatrophin* gene is located on chromosome 19p13.2, a locus associated with serum high-density lipoprotein (HDL) levels [6,7]. The *betatrophin* gene is embedded on the strand opposite that of the host gene dedicator of cytokinesis 6 (*DOCK6*), implying tissue-specific expression (Figure 1A) [8]. The mouse betatrophin gene has been identified as the *Gm6484* gene positioned on chromosome 9. Moreover, the low-density lipoprotein receptor (*LDLR*) gene is located in close proximity to the *betatrophin* gene (within a distance of approximately 150 kb) in both human and mouse. This phenomenon further emphasizes the association of the 19p13.2 locus and serum HDL levels. The human *betatrophin* transcript (NM_018687, Figure 1B) comprises four exons encoding a protein of 198 amino acids (NP_061157, human betatrophin in Figure 2). Betatrophin and its orthologs have been detected in the class Mammalia, but neither the transcript nor its polypeptide homologs are present in birds, amphibians, insects or vertebrate fish species. This particular expression of betatrophin in mammals implicates a physiological role in contributing to the characteristics unique to mammals, such as homeothermy, pregnancy and lactation. No known conserved domains have been identified in betatrophin, and the nearest paralog is angiopoietin-like protein 3 (ANGPTL3), which shares 22% protein identity (ANGPTL3, AAH07059.1). Intriguingly, similar to *betatrophin*, the *ANGPTL3* gene is embedded on the strand opposite that of the host gene dedicator of cytokinesis 7 (*DOCK7*) in chromosome 1. The comparable gene structures suggest that betatrophin and ANGPTL3 are derived from ancestral gene duplication [9]. Protein sequence alignment of betatrophin orthologs in mammals revealed that several regions are partially conserved (Figure 2). Sequence analysis further disclosed that the *N*-terminal region of betatrophin contains signal sequences for secreted or membrane-bound protein [10]. In addition, several predicted protein modification sites exist in the *N*-terminal region of betatrophin (estimated using Pro-site), among them, three *N*-myristoylation sites, implying that lipidated betatrophin is lipophilic and may be anchored to the membrane structure. The presence of predicted casein kinase phosphorylation sites further suggests that functional regulation of betatrophin activity participates in a rapid signal response. These hypothetical modification sites appear highly conserved across the species, from wallaby to human. In general, betatrophin sequences are highly conserved at the *C*-terminal end and those of several species (gorilla, sheep and hyrax) bear additional conserved *N*-terminal regions. To date, the conservation of betatrophin protein sequence has highlighted a critical role. However, further research is required to determine the significance of specific betatrophin motifs in mammals.

## 3. Cellular Localization of Betatrophin

Several researchers have investigated the subcellular localization of betatrophin, with the aim of gaining insights into its biological activities.

### 3.1. Vesicular Betatrophin

In hepatoma cells, betatrophin is mainly localized in the cytoplasm with vesicle-like distribution [11]. Several patterns of betatrophin vesicles with variable sizes have been detected [11]. The small dot-like betatrophin vesicles (≤1 μm) are usually solid and dispersed in the cytoplasm. The larger betatrophin vesicles (1–2 μm) become empty and are often associated with lysosome-associated membrane protein 2 (LAMP2) and/or lipid droplet protein perilipin2 (PLIN2), suggesting the involvement of betatrophin in hydrolysis degradation or the lipid regulation pathway. Occasionally, betatrophin vesicles are clumped together and adhered to the large LAMP2 vacuoles (2–10 μm), indicating that a proportion of betatrophin is functionally associated with large multivesicular bodies (MVBs) [11]. These phenomena were further demonstrated with organelle density fractionation data showing that betatrophin co-fractionates with light PLIN2 and heavy LAMP2, consistent with its cellular localization [11]. Given that several potential *N*-myristoylation sites are highly conserved within betatrophin, further molecular research is required to address the association between *N*-myristoylation and cellular localization.

### 3.2. Secretion of Betatrophin

The *N*-terminal sequence (1–20 amino acids) of betatrophin contains a predicted signal peptide, suggesting that the protein is secreted or membrane-bound [10]. Serum betatrophin has been detected in humans and mice, and betatrophin secretion levels are usually correlated with serum TG or (very low-density lipoprotein) VLDL levels (discussed in detail below) [2,3,4,5,12,13,14,15]. Intracellular betatrophin is associated with lipid droplets, implying that betatrophin may serve as a lipoprotein and might be secreted or taken up with a lipid-associated compartment [11].

## 4. Expression of Betatrophin

Secreted betatrophin protein is distributed into most tissues by the circulation. However, increasing evidence indicates that betatrophin mRNA is originally expressed in liver, white adipose tissue (WAT) and brown adipose tissue (BAT) [2,3,4]. Although the biological roles of betatrophin remain to be fully established, the causes of expression and physiological profiles determined so far suggest that the protein is associated with intrinsic energy intake and expenditure, particularly for lipid metabolism (Table 1, Table 2 and Table 3).

**Table 1:** Clinical correlation of betatrophin in human.

**Detection Model**	**Analysis Factor**	**Correlation**	**Detection Method**	**Reference**
	Age	+	ELISA (EIAAB)	[16]
	Obese/overweight	+	ELISA (Phoenix)	[17]
	Morbidly obese	NC	ELISA (EIAAB)	[12]
	BMI	+	ELISA (Phoenix)	[17]
	BMI	−		[17]
	HDL-/LDL-cholesterol	−		[2]
**Type I Diabetes**	Type I Diabetes	+	ELISA (EIAAB)	[13]
	Cholesterol in TID	NC	Sequencing (R59W variant)	[13]
	Triacylglycerol in TID	NC	ELISA (EIAAB)	[13]
**Type II Diabetes**	Type II Diabetes	+	ELISA (EIAAB) ELISA (Phoenix)	[14,16,18]
	Type II Diabetes	−	ELISA (Cusabio)	[19]
	Type II Diabetes	NC	ELISA (EIAAB)	[12]
	Glucose in TIID	+	ELISA (Phoenix)	[14]
	Glucose in TIID	NC	ELISA (EIAAB)	[12]
	Insulin in TIID	+	ELISA (Phoenix)	[14]
	Insulin in TIID	NC	ELISA (EIAAB)	[12]
	Hemoglobin A1c	+	ELISA (EIAAB)	[16]
	BMI in TIID	+	ELISA (Phoenix)	[14]
	BMI in TIID	−	ELISA (Cusabio)	[19]
	BMI in TIID	NC	ELISA (EIAAB)	[12]
	Triacylglycerol in TIID	NC	ELISA (Phoenix)	[14]
	Total cholesterol in TIID	+	ELISA (EIAAB)	[12]
	Total cholesterol in TIID	NC	ELISA (Phoenix)	[14]
	HDL cholesterol in TIID	+	ELISA (Cusabio)	[19]
	HDL cholesterol in TIID	NC	ELISA (Phoenix)	[14]
	LDL cholesterol in TIID	+	ELISA (EIAAB)	[12]
	LDL cholesterol in TIID	NC	ELISA (Phoenix)	[14]
	Apolipoprotein B in TIID	+	ELISA (EIAAB)	[12]

BMI, Body mass index; TID, Type I diabetes; TIID, Type II diabetes; ELISA, Enzyme-linked immunosorbent assay; +, Positive correlation; −, Negative correlation; and NC, No correlation.

**Table 2:** Regulation of betatrophin expression.

Regulator	Treatment	Expression/Localization	Model Organism	Reference
**Positive Regulator**				
Nutrition intake	Caloric intake	Protein in serum	Human	[14]
	Caloric intake	mRNA	Human adipocytes	[20]
	High fat diet	mRNA in liver, BAT and WAT	Mouse	[2,4,9]
Insulin	Insulin	mRNA	Mouse 3T3 and Human adipocytes	[3]
Insulin antagonist	S961	mRNA in liver and WAT	Mouse	[5]
Thyroid hormone	Thyroid hormone	mRNA and protein	Human HepG2 cell	[11]
Irisin	Humanirisin	mRNA	Mouse 3T3	[21]
Cold stimulation	4 °C for 4 h	mRNA in BAT	Mouse	[9]
Gestation	Gestation	mRNA in liver	Mouse	[5]
SREBP1a/SREBP2	Transgenic mice	mRNA in liver	Mouse	[2]
Liver X receptor agonist	T0901317	mRNA in liver	Mouse	[2]
**Negative Regulator**				
Fasting	Fasting	mRNA in liver, BAT and WAT	Mouse	[2,4,9]
TNFα	TNFα	mRNA	Mouse 3T3	[3]
Lypolysis inducer	db-cAMP, forskolin, Isoproterenol	mRNA	Mouse 3T3	[3]

**Table 3:** Functional characterization of betatrophin.

Analyzed Result	Manipulation of Betatrophin Expression			Model Organism
	Overexpression	Null Mice	Knockdown	
β-Cell proliferation	↑ [5]	ND	ND	Mouse
Insulin production	↑ [5]	NS	ND	Mouse
Blood glucose	↓ [5]	NS	ND	Mouse
TG	Serum TG ↑ [2,4] HepG2 TG ↓ [11]	Serum TG ↓ [3,15] Hepatic TG N.S. [15]	3T3 Adipocytic TG ↓ [3] HepG2 TG ↑ [11]	Mouse, 3T3 and HepG2 cell
ANGPTL3	Cleavage ↑ [2] ANGPTL3 level ↓ [2]	ANGPTL3 level ↑ [15]	ND	Mouse
Autophagy flux	↑ [11]	ND	↓ [11]	HepG2 cell
Serum LPL activity	↓ [4]	↑ [15]	ND	Mouse
Mice body weight	ND	↑ [15]	ND	Mouse
Mice fat mass	ND	↓ [15]	ND	Mouse
NEFA	ND	↓ [15]	ND	Mouse
VLDL-TG uptake	ND	In WAT ↓ [15]	ND	Mouse

↑, Increased; ↓, Decreased; N.S., No significant change; ND, Not determined.

### 4.1. Physiological Expression

*Betatrophin* mRNA is expressed at high levels in mouse liver and brown fat, and moderately in subcutaneous fat, perigonadal fat, kidney, small intestine and heart [3,4]. In humans, *betatrophin* mRNA is almost uniquely expressed in liver, with limited expression in fat, brain, rectum and heart [2,3,4]. This tissue-specific expression corresponds to previous categorization of nest genes (Figure 1A) [8]. Interestingly, the *betatrophin* mRNA expression profile is concordant with that of α-fetoprotein (AFP) in several liver cancer cell lines [22]. Detection of hypermethylated loci of the *betatrophin* gene in cirrhotic liver and hepatocellular carcinoma implies that *betatrophin* transcriptional activity may not be required for hepatic tumorigenesis [23]. In other words, *betatrophin* expression also reflects normal differentiated liver.

Recent reports have shown that a *betatrophin* transcript variant and expression levels are associated with clinical or pathological symptoms (Table 1). For instance, the genetic R59W variant of *betatrophin* is associated with reduction of HDL-C and LDL-C in African American and Hispanic participants [2]. Serum levels of betatrophin protein are increased in type I diabetes patients, but display no obvious correlation with cholesterol or triacylglycerol [13]. Recently, the increased circulating levels of betatrophin protein in type II diabetes patients have been identified via three independent groups [14,16,18]. Fu and colleagues demonstrated that serum betatrophin protein levels are elevated in type II diabetes and overweight/obese groups, compared to non-diabetic and lean control subjects, respectively [14]. Furthermore, serum betatrophin protein was positively correlated with fasting glucose, insulin and BMI in patients with type II diabetes but not with levels of triacylglycerol, total cholesterol, HDL cholesterol and LDL cholesterol [14]. Furthers, Espes *et al.* [16], observed betatrophin protein levels are positively correlated with age in the health control and with hemoglobin A1c in the type II diabetes patients. On the other hand, Gómez-Ambrosi *et al*. [19], disclosed that serum betatrophin levels are decreased in obese participants and Type II diabetes patients. They further demonstrated that serum betatrophin protein levels are negatively correlated with BMI values, and positively correlated with insulin sensitivity and HDL cholesterol [19]. Also, Fenzl and colleagues showed no significant differences in serum betatrophin protein levels between lean and morbidly obese or between non-diabetic and type II diabetes patients [12]. Nevertheless, betatrophin protein levels were positively correlated with total cholesterol, LDL cholesterol and apolipoprotein B in subjects with morbidly obese and type II diabetes, although no association with glucose homeostasis parameters (glucose, insulin and BMI) was observed [12]. There are conflicting results of serum betatrophin levels in diabetes patients, and the associations of betatrophin levels with BMI values, lipid profiles or insulin sensitivity are controversial [12,13,14,16,18,19]. Notably, the detection of serum betatrophin levels in these works was determined using antibody-based ELISA, which means the choice of antibodies may lead to discrepant results. Very recently, Fu *et al*. [17], using different ELISA kits (EIAAB, E1164H and Phoenix, EK-051-55), provided evidence that most of the circulated betatrophin proteins lack an *N*-terminus. The antibodies used in the EIAAB kit recognize the *N*-terminus of betatrophin and those in the Phoenix kit recognise the *C*-terminus. Fu and colleagues suggest that full length betatrophin protein in serum may undergo proteolytic cleavage and the residual *C*-terminal fragment of betatrophin was released [17]. More importantly, the betatrophin levels determined by ELISA kits of Phoenix and EIAAB are positively and negatively correlated with the BMI values, respectively [17]. In addition, the activation of betatrophin expression might coordinate with other factors. For instance, glucose is required for insulin-induced result of various demographic characteristics (age, weight and BMI *etc*.) between those study populations. Despite that betatrophin appears to be correlated with specific blood lipids, further extensive studies are still required to comprehensively establish the biological significance of betatrophin in circulating lipids.

### 4.2. Nutrition-Induced Betatrophin

In a study screening caloric intake-related genes in human adipocytes, both *betatrophin* and *DOCK6* mRNA expression levels were elevated upon reintroduction of ordinary foods in the obese group previously administered a low-calorie diet and in the healthy group subjected to overfeeding [20]. Upon administration of a high-fat diet in mice, *betatrophin* mRNA was induced in liver, BAT and WAT tissues, whereas *betatrophin* was suppressed in fasting mice [2,4,9]. Furthermore, following a period of food deprivation, refeeding led to enhanced *betatrophin* mRNA expression within 8–12 h [2,3]. The serum levels of betatrophin protein were increased within 2 h following a defined meal in 12 non-diabetic human individuals [14]. These findings provide evidence that betatrophin mRNA and protein levels are associated with nutritional intake. Thus, betatrophin expression levels may fluctuate with experimentally induced food intake behavior. Fu and co-workers further demonstrated that the betatrophin expression profile is distinct from that of other angiopoietin-like proteins, signifying that betatrophin is a novel nutrition-regulated gene that performs a different biological role compared to other ANGPTL family proteins [9].

### 4.3. Hormonal Regulation of Betatrophin

#### 4.3.1. Insulin

Ren and colleagues initially reported upregulation of *betatrophin* mRNA in a 3T3-L1 preadipocytes differentiation model [3]. Based on subsequent studies on nutritional regulation, the group proposed that betatrophin is a novel regulator of lipid metabolism. They found that, during insulin-induced fat lipogenesis, *betatrophin* transcripts were also induced in mouse 3T3 and human adipocyte cells. Interestingly, insulin-induced betatrophin expression was obligatory in the presence of glucose. Once 3T3-L1 was separately maintained in glucose or insulin, no significant induction of betatrophin was evident [3]. This finding suggests the double-checked and crosstalk between glucose and insulin stimuli are necessary for betatrophin induction. The group of Yi *et al*. [5], demonstrated that S961, a 43 amino acid peptide that binds the insulin receptor, specifically induces betatrophin expression in liver and white fat. Further investigation led to the conclusion that S961 increases insulin levels through betatrophin and mediates pancreatic cell regeneration. Notably, the S961 concentrations of 5–20 nmol/week used by Peng were sufficient to antagonize the insulin receptor *in vitro* and *in vivo* [24]. However, S961 also showed insulin agonist activity at concentrations of 1–10 nM [25]. Importantly, another study demonstrated that while S961-treated rats exhibit hyperinsulinemia, hyperglycemia and insulin resistance, S961 treatment reduces BAT and WAT adipocyte sizes as well as hepatic glycogen [26]. This finding is inconsistent with other results showing that betatrophin expression is not necessarily positively correlated with lipid content or lipogenesis activity. Furthers, Yi *et al*. [5], observed betatrophin induction during the gestation stage, which displays accelerated β-cell replication [27]. Serum levels of TG and non-HDL cholesterol have been shown to be increased in late pregnancy mice whereas the HDL cholesterol level is reduced [28]. In humans, TG, total/HDL/LDL cholesterol contents are further increased at different periods of gestation [29]. Normal gestation shifts the LDL profile towards the smaller, denser lipid species, which are more susceptible to oxidation and lipolysis [30,31,32]. The results collectively indicate that betatrophin expression induced by insulin is either directly triggered by elevated mRNA transcription or indirectly coordinated with other insulin-mediated processes.

#### 4.3.2. Thyroid Hormone

The thyroid hormone (TH) mediates cell growth, differentiation and homeostasis by binding to the nuclear thyroid hormone receptor. Various regulatory pathways involving TH have been characterized within distinct tissues, stages and species [33,34]. For maintenance of hepatic lipid homeostasis, the thyroid hormone directs regulation or crosstalk with nutrient-activated nuclear receptors to regulate lipid-associated gene transcription [35,36,37]. Intriguingly, on the one hand, thyroid hormone promotes lipid catabolism through decreasing the total amount of cholesterol, low-density lipoproteins, and chylomicron particles [36,38]. On the other hand, T3 induces upregulation of several lipogenic genes, including acetyl-CoA carboxylase, FAS (fatty acid synthase), and NR1H3 (nuclear receptor subfamily 1, group H, member 3/liver X receptor-α), promoting lipid biosynthesis [39,40]. Notably, T3 also induces upregulation of several lipid metabolic genes, including low-density lipoprotein receptors, CYP7A1 (cytochrome P450, family 7, subfamily A, polypeptide 1/cholesterol-7α hydroxylase), and LIPC (lipase, hepatic) [41,42,43]. Although the thyroid hormone stimulates lipogenesis in experimental models, decrease in triglycerides, hepatic triglycerides and VLDL is simultaneously observed [44,45]. Thus, other thyroid hormone activities, such as increased fatty acid (FA) oxidation, may additionally contribute to lipid clearance [11,44].

A previous report by our group revealed that *betatrophin* mRNA is induced by the thyroid hormone in HepG2 cells [11]. Subsequent studies confirmed that transcriptional regulation is dependent on the thyroid hormone receptor that binds to the *betatrophin* upstream element. *Betatrophin* is a novel gene dramatically activated by the thyroid hormone (top 5% ranking), and therefore of significant interest to our group. Interestingly, although the thyroid gland is present in all vertebrates, thyroid hormones affect metabolic rates and thermogenesis only in homoeothermic species [46,47,48,49]. Such a role appears to be acquired during late evolution, highlighting the phylogenetic character of *betatrophin* gene evolution in mammals. Our experiments further showed that T3-induced betatrophin is further elevated by ammonium chloride, a weak base lysosomotropic alkalinization agent, implying that a proportion of betatrophin is degraded through the endosomal/lysosomal pathway [50].

### 4.4. Other Factors Modulating Betatrophin Expression

A number of additional factors that mediate betatrophin expression have been identified. Recently, Zhang *et al*. [21], reported that irisin, a newly defined peptide encoded by the fibronectin type III domain-containing 5 (FNDC5) gene, induces betatrophin expression. Interestingly, although betatrophin is suppressed by the lipolysis inducer, forskolin-induced BAT lipolysis is further elevated in the presence of thyroid hormone and irisin [51,52]. Fu *et al*. [9], demonstrated that low temperature exposure (4 °C for 4 h) induces betatrophin expression but suppresses ANGPTL4/2 expression in mice BAT. The inverse responses of betatrophin and ANGPTL proteins to cold, high-fat diet (HFD) or fasting indicates opposite physiological roles of betatrophin and the ANGPLT family to some extent [53,54]. Correspondingly, the thyroid hormone activates betatrophin expression but suppresses ANGPTL3 in liver cells, despite the finding that betatrophin also interacts with ANGPTL3 and promotes its cleavage [2,11,55]. Sterol regulatory element-binding proteins, SREBP1a and SREBP2, specifically activate betatrophin expression as well as FAS and HMGR (3-hydroxy-3-methylglutaryl-CoA reductase or HMG-CoA reductase) in transgenic mice liver [2]. Similarly, the liver X receptor agonist, T0901317, induces betatrophin and FAS expression in mouse liver [2]. The lipolysis activators, TNFα, dibutyrl cAMP (db-cAMP), forskolin (Forsk), and isoproterenol (Iso), suppress the betatrophin transcript level in adipocytes [3]. In conjunction with the finding that thyroid hormone and irisin induce betatrophin, these results suggest that betatrophin is simultaneously involved in lipolysis and lipogenesis within liver, BAT and adipocytes. Other gene expression profile studies have further disclosed that betatrophin is regulated by several stimuli, including HCV-1b, interferon-α, cationic amphiphilic drugs and trichostatin A, implying functions in the maintenance of metabolic homeostasis and regulation of cellular stress response in liver [56,57,58,59].

## 5. Functional Characterization of Betatrophin

Betatrophin expression levels may be relevant to glucose/lipid homeostasis *in vitro* and *in vivo*. The activities of betatrophin in these processes have been further clarified through manipulation of betatrophin expression (Table 3).

### 5.1. Alterations in Lipid Levels

Earlier studies demonstrated that mouse body weight and fat mass as well as serum triglycerides and NEFA (non-esterified fatty acid) levels are reduced in betatrophin-null mice whereas serum cholesterol, plasma glucose (fasted and re-fed) and insulin levels are not significantly altered, compared with wild-type littermates [3,15]. The main decrease in triglyceride species was derived from the VLDL fraction [15]. Serum triglycerides were particularly reduced in betatrophin-null mice after re-feeding. In contrast to serum triglyceride levels, hepatic triglyceride levels were not reduced in betatrophin-null mice. [15]. Mice lacking betatrophin expression specifically induce plasma lipase activity whereas they suppress VLDL-TG uptake in WAT after re-feeding [15]. Furthermore, although serum betatrophin levels are associated with atherogenic lipid profiles, betatrophin-null and wild-type mice were similar in terms of VLDL-TG uptake in heart with fasting or re-feeding regimens [15]. Upon knockdown of betatrophin, the intracellular TG content was decreased in 3T3-L1 adipocyte cells, but increased in HepG2 hepatic cells [3,11].

Zhang and co-workers showed that adenovirus-mediated betatrophin expression in mice liver enhances serum TG levels [4]. Additionally, recombinant betatrophin proteins expressed in *E. coli* lacking eukaryotic modifications were sufficient to inhibit LPL activity [4]. Quagliarini and co-workers further clarified that the betatrophin-induced serum TG content is ANGPTL3-dependent and co-expression of betatrophin with ANGPTL3 further increases the serum TG level [2]. Interestingly, overexpression of betatrophin in ANGPTL3-deficient mice conversely reduced the serum TG, cholesterol and NEFA levels [2]. These results suggest that ANGPTL3 levels are critical for the switch of betatrophin function. Moreover, thyroid hormone-induced betatrophin-mediated lipolysis, in concert with the thyroid hormone suppresses ANGPTL3 activity [11,55].

### 5.2. Correlation of Betatrophin and ANGPTL3 Functions

Due to homolog and functional similarities of betatrophin and ANGPTL3, several groups have focused on their relevance. Expression of the full-length and cleaved forms of ANGPTL3 was elevated in betatrophin-null mice, implying that betatrophin suppresses endogenous ANGPTL3 expression and betatrophin expression is not required for ANGPTL3 cleavage [15]. Elevated ANGPTL3 expression was inconsistent with the finding that lipase is activated in betatrophin-null mice [15]. Conversely, transduction of recombinant adenovirus expressing betatrophin in mouse liver reduced serum ANGPTL3 protein levels but had no influence on mRNA levels [2]. In addition, both full-length and *N*-terminal fragments of ANGPTL3 were immunoprecipitated with *C*-terminal FLAG-tagged betatrophin in mice plasma [2]. These findings not only signify physical interactions between betatrophin and ANGPTL3 but also indicate that betatrophin is a secreted protein and the native *C*-terminal end is accessible to the anti-FLAG antibody. However, overexpression of betatrophin in HepG2 cells paradoxically promoted soluble ANGPTL3 cleavage in the medium [2]. Thus, distinct regulatory mechanisms between betatrophin and ANGPTL3 may exist under different conditions.

### 5.3. Betatrophin Regulates Autophagic Flux

Previous reports showed that both betatrophin mRNA and protein are stimulated by the thyroid hormone in liver cells. In addition, a vesicle-like pattern of betatrophin was observed around lipid droplets or within the lysosome-associated compartment in cells, implying its relevance in intracellular metabolism [11]. Betatrophin overexpression specifically activated autophagic flux and lipid metabolism, as evident from lipidated LC3 (LC3-II) and oxygen consumption rate, respectively, in our experiments. Moreover, betatrophin overexpression increased the acidic compartment while reciprocally reducing hepatic lipid droplets. Interestingly, T3-induced betatrophin and lipid content further accumulated upon treatment with ammonium chloride, an autolysosome maturation inhibitor, providing evidence that a proportion of betatrophin and lipid droplets are degraded in the autolysosome. These results collectively suggest that betatrophin regulates lipid metabolism through a lysosome-mediated autophagic process.

As mentioned previously, betatrophin levels are regulated by various factors, including caloric intake, insulin, irisin, cold, HCV-1b, interferon-α, cationic amphiphilic drugs and trichostatin A [3,9,20,21,56,57,58,59]. These targeting factors have been shown to be involved in the lysosomal or autophagy pathway. For instance, extended longevity via caloric restriction and resveratrol is associated with autophagy induction [60,61]. Insulin-stimulated VLDL-apoB100 degradation in mouse primary hepatocytes through autophagy has been shown to be ATG5-dependent [62]. Irisin, activated by exercise as well as PGC-1α overexpression, potently induces brown/beige fat gene expression [51]. Interestingly, PGC-1α expression is induced by the thyroid hormone and cold exposure, and exercise and cold-induced autophagy have been reported [63,64,65,66]. Hepatitis C virus infection and interferon-α response induce autophagy whereas cationic drugs inhibit lysosomal activity and trigger autophagic vacuolization [67,68,69]. Conversely, TSA (trichostatin A) specifically impairs histone deacetylase activity and transverse aortic constriction-induced autophagy [70]. It is possible that these agents act through betatrophin, utilizing autophagic activity to modulate lipid turnover, which would explain the correlation between betatrophin expression and lipid profiles.

### 5.4. Betatrophin Induces Proliferation of Pancreatic Cells

Yi and colleagues focused on the insulin receptor antagonist, S961, which induces hyperglycemia, hyperinsulinemia and glucose intolerance in mice [5,26]. Microarray analysis showed that betatrophin is potently induced by S961 in mouse liver and white adipocytes. The group further detected the secreted form of betatrophin, as observed from ectopic expression, in the culture supernatant and plasma of Hepa 1–6 and liver cells, respectively. More importantly, overexpression of betatrophin in mouse liver significantly induced pancreatic β-cell proliferation, mass expansion and insulin production. The percentages of dividing cells were markedly enhanced by betatrophin in pancreatic cells, whereas no significant alterations were observed in liver, WAT or BAT cells. Betatrophin also promoted increments of the Ki67-positive signal and the proliferation activators cyclin A1, cyclin F and E2F2, while inhibiting the suppressors cdkn1a and cdkn2a in pancreatic islet cells. Betatrophin-overexpressing mice showed lower blood glucose and elevated fasting insulin in plasma. In contrast to S961, insulin resistance was not induced by betatrophin overexpression. Taken together with previous findings, insulin-induced betatrophin might not act through insulin receptor. At the high level of serum glucose, insulin promotes betatrophin expression in the presence of glucose. Elevated betatrophin protein further activates β-cell proliferation and insulin production which promotes glucose uptake in storage cells. Once the glucose levels are decreased and insufficient for insulin-induced betatrophin, positive feedback was blocked. On the other hand, hyperglycermia was induced by S961-inhibited insulin receptor and glucose uptake. Thus, insulin-induced TD26 and consequent β-cell proliferation and insulin production would not cease under hyperglycemia. These findings may explain why insulin resistence was induced by S961, but not induced by betatrophin. Thus, betatrophin may provide a novel therapeutic approach for the treatment of diabetes through pancreatic cell regeneration. In contrast to the marked increase in betatrophin-induced β-cell DNA replication in mouse, Yang and co-workers recently reported that transplanted human β-cells are completely unresponsive to S961 treatment [71]. Stewart *et al*., further highlighted that conflicting regulation of β-cell replication between humans and rodents is not uncommon [72]. While several protein regions are conserved in mammalian betatrophin orthologs, many tiny and discontinuous regions vary in primates and rodents, indicative of different corresponding receptors or modulators.

## 6. Conclusions

**Figure 3:**
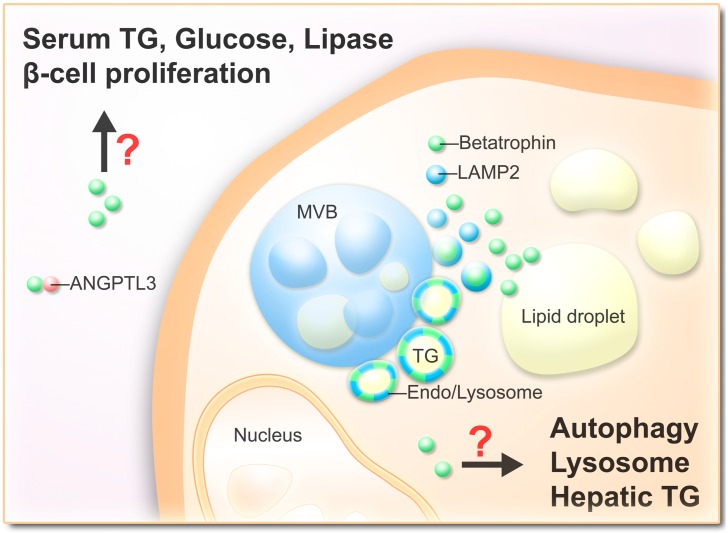
Schematic representation of hypothetic betatrophin functions. Secreted betatrophin interacts with ANGPTL3 and/or modulates β-cell proliferation, serum TG levels, serum glucose levels and lipase activity. Intracellular betatrophin associated with lipid droplets and endosome/lysosome vesicles which may serve as a lipoprotein and activate autophagy.

Based on previous and current research, the roles of betatrophin in lipid metabolism have been highlighted. However, further issues are yet to be clarified, for instance, whether betatrophin serves as a lipoprotein that is embedded with lysosome/lipid-associated vesicles and secreted in plasma, and the physiological benefits and cell functions promoted by betatrophin expression (Figure 3). Most importantly, the issue of whether serum lipid or β-cell replication profiles can be altered in human species via manipulation of betatrophin expression remains to be resolved. Further studies are warranted to comprehensively evaluate the effects of betatrophin before its application as a therapeutic agent for metabolic syndrome.

## Abbreviation

AFP: α-fetoprotein
ANGPTL3: angiopoietin-like protein 3
ANGPTL8: angiopoietin-like protein 8
BAT: brown adipose tissue
C19orf80: chromosome 19 open reading frame 80
DOCK6: dedicator of cytokinesis 6
DOCK7: dedicator of cytokinesis 7
FAS: fatty acid synthase
FNDC5: fibronectin type III domain-containing 5
HDL: high-density lipoprotein
HMGR: 3-hydroxy-3-methylglutaryl-CoA reductase
LAMP2: lysosomal-associated membrane protein 2
LDs: lipid droplets
LDLR: low-density lipoprotein receptor
MVB: multivesicular body
PLIN2: perilipin2
RIFL: refeeding induced fat and liver
T3: thyroid hormone 3,3',5-triiodo-l-thyronine
TG: triglyceride
WAT: white adipose tissue
